# Which?

**Published:** 1882-06

**Authors:** 


THE
### HOMŒOPATHIC PHYSICIAN.
A MONTHLY JOURNAL OF MEDICAL SCIENCE.

"If our school ever gives up the strict inductive method of Hahnemann, we are lost, and deserve only to be mentioned as a caricature, in the history of medicine."—CONSTANTINE HERING.

Vol. II. JUNE, 1882. No. 6.

### EDITORIAL.

WHICH?—We clip the following denunciation of Homœopathy from an exchange. The writer, we may as well explain, pretends to be a follower of Hahnemann!

This homœopath has discovered what no man before him ever knew:—that there can be *two* laws of *nature*, which oppose and contradict one another! Thus does he announce his grand discovery:

"There are two main principles in therapeutic science, *contraria* and *similia*. Both are natural. The principle of contraries has been applied in practice more than three thousand years; it is as just as *natural* as the other, the principle of similars, of more recent history."

Newton or Hahnemann is far behind this great discoverer, for they each discovered only one law of nature! Yet this genius, not satisfied with his two laws, proceeds thus to demolish Hahnemann's one: *

"It appears to be evident that the high-potency party have held sway too long. They represent a form of medical spiritualism which is unsound in theory and very prejudicial to the interests of true Homœopathy. Notwithstanding this, they are holding prominent positions in all our medical colleges and societies, and at the same time are indorsing and advocating extravagant theories which are evidently subversive of the fundamental principles of Homœopathy. They have held these positions so long that they have evidently come to the belief that they alone represent Homœopathy; hence, by right, are privileged to dictate to the low-potency party regarding all matters involving homœopathic interests. They appear to be oblivious of, or at least ignore the fact, that this nondescript method of practice is repudiated by many of the best and wisest men in our school. They do not yet appear to

* We here class the "high-potency party" as representatives of Hahnemann's law, because they chiefly represent that law.

comprehend the fact that the recognition and advocacy of the false theory of dynamization *must cease;* not because the low-potency party desire its dissolution—but because it is the embodiment of error and, from the homœopathic point of view, of error *only*.

"This hypothetical method of practice has had its ephemeral existence, as chiefest of 'medical illusions,' and has been discarded by a large proportion of the membership of the homœopathic school. Every day that we allow this empirical method to be taught at our medical colleges, we are *acting a lie!* Every day that we listen to reports of these nondescript dynamic cases, at the meetings of our societies, and publish them as homœopathic, without protest, we are *acting a lie!* In the interests of *truth*, therefore, the work of elimination will go forward.

"It is well that the attention of our school is being called to this important subject, and it is desirable that measures be inaugurated for the removal of all professors in our colleges who represent these obnoxious doctrines, and the appointment in their places of others who will teach sounder and more rational principles.

"Dr. H. W. Taylor says: 'This small moiety infest our colleges. They do not teach homœopathic therapeutics. Hence, there is a growing demand that they be retired, and Low Dilution homœopaths be put in their places.'*

"It will be useless to attempt the changes in the faculties of our medical colleges until after this singular form of medical error has been openly repudiated by the homœopathic school. As the sentiments that are approved at meetings of our large associations reflect, with a good degree of accuracy, the views of a majority of the profession, the wisest course that I can suggest is, the adoption, by our State and local societies, of a declaration to the effect that all practice with potencies higher than the 12th be classed as dynamic. To this no one can reasonably object. It does not in the least interfere with the rights or privileges of the members.† It merely places, for future observation and analysis, the results of this evidently non-homœopathic method of practice in a department by itself.

"This once accomplished, thereafter true Homœopathy will not be cumbered by the humiliating association with that which is purely fictitious, visionary

---

* *New York Medical Times*, Dec., 1881, page 287.

† The *naiveté* of this gentleman is truly refreshing. See his reasoning. He says: Let us declare that all practice with potencies above the 12th is dynamic. Reporting "nondescript dynamic cases" is "*acting a lie.*" To this declaration (*i. e.*, classing potencies above the 12th dynamic) "no one can reasonably object!!"

So according to this innocent-low-potency-true-homœopath no man should "reasonably object" to being classed as a "*liar.*" Others are probably more sensitive for their fair name than the Albany statesman-physician.

By what scientific reasoning does this *savant* declare that the limit of potentiation is reached at the 12th? Does he arrogate unto himself the power of God, and say: Thus far and no farther shalt thou go? Or does he say: Let my laziness and incompetency be the limit beyond which science cannot go?

and hypothetical. The question of the *small dose* (not the minimum dose, which has been the principal disturbing element in our school), will then assume much more manageable proportions."

We quote these passages, not from any great importance the writer possesses, for he is merely a straw, floating on the stream of mongrel eclecticism; but since straws show the direction of the current, it behooves true homœopathists to take note of them. When such twaddle can be openly published and circulated as genuine Homœopathy is it not time for the Hahnemannians to be *actively* working for their science? Is it not their *duty*, and should it not be their pleasure to do so? Passive adherence—*i. e.*, work in the office and sick-room—to Hahnemannism is very good; but something more is needed at this time. Each Hahnnemannian practitioner should join the I. H. Association and work for his science.

The writer of the above-quoted passages endeavors to make believe that the issue between homœopathists and eclectics is simply one of dose; that high potency and low potency are synonyms for homœopathists and eclectics. This he knows to be false. Many low-potency men are as good homœopathists as the advocate of the M or CM. For it is the manner in which one prescribes, not the dose used, which makes him an homœopathist or an eclectic.

From what we have said in our April issue and in the above, we have endeavored to set plainly before the profession the fact that there are two distinct parties in the so-called homœopathic school. The one representing eclectic methods and practice; the other, the principles and practice of Hahnemann. The time has now come when all practitioners must choose which party they will aid and assist. Will you retrogade or advance; be an eclectic or an homœpathist?

